# Time-varying and dose-dependent effect of long-term statin use on risk of type 2 diabetes: a retrospective cohort study

**DOI:** 10.1186/s12933-020-01037-0

**Published:** 2020-05-16

**Authors:** Eonji Na, Sunyoung Cho, Dae Jung Kim, Junjeong Choi, Euna Han

**Affiliations:** 1 Department of Pharmacy and Yonsei Institute of Pharmaceutical Sciences Research, College of Pharmacy, Yonsei University, 162-1 Songdo-dong, Yeonsu-gu, Incheon, South Korea; 2 Department of Pharmaceutical Medicine and Regulatory Sciences, College of Medicine and Pharmacy, Yonsei University, Incheon, South Korea; 3 Integro Medi Lab Co., Ltd., Seoul, South Korea; 4 Department of Endocrinology and Metabolism, Ajou University School of Medicine, Suwon, South Korea

**Keywords:** Statin, Type 2 diabetes, Time-varying survival analysis

## Abstract

**Background:**

We evaluated the effect of statin use on new-onset type 2 diabetes among individuals without atherosclerotic cardiovascular disease (ASCVD) using nationally representative South Korean claims data (2002–2013, N = 1,016,820).

**Methods:**

A total of 13,698 patients (statin users 5273, non-statin users 5273) aged 40–74 years, newly diagnosed with dyslipidemia but without any history of diabetes or ASCVD, were selected in 2005. We followed up the final sample until 2013 and evaluated the cumulative incidence of type 2 diabetes. We used extended Cox regression models to estimate the time-varying adjusted hazard ratios of statin use on new-onset type 2 diabetes. We performed further analyses based on the cumulative defined daily dose of statin received per year to evaluate the degree of risk compared to non-statin users.

**Results:**

Over the mean follow-up period of 7.1 years, 3034 patients developed type 2 diabetes; the number of statin users exceeded that of non-users, demonstrating that statin use significantly increased the risk of new-onset type 2 diabetes. The risk of new-onset type 2 diabetes differed among statin users according to cDDD per year (adjusted HR = 1.31 [95% CI 1.18–1.46] for less than 30 cDDD per year; 1.58 [1.43–1.75] for 30–120 cDDD per year; 1.83 [1.62–2.08] for 120–180 cDDD per year; and 2.83 [2.51–3.19] for more than 180 cDDD per year). The diabetogenic effect of pitavastatin was not statistically significant, but the risk was the largest for atorvastatin. Long-term exposure (≥ 5 years) to statins was associated with a statistically significant increase in the risk of new onset type 2 diabetes in all statin subtypes explored, with the highest magnitude for simvastatin (HR = 1.916, 95% CI 1.647–2.228) followed by atorvastatin (HR = 1.830, 95% CI 1.487–2.252).

**Conclusions:**

Statin use was significantly associated with an increased risk of new-onset type 2 diabetes. We also found a dose–response relationship in terms of statin use duration and dose maintenance. Periodic screening and monitoring for incident type 2 diabetes may be warranted in long-term statin users.

## Background

Statins, originally used in the treatment of dyslipidemia, are now the drugs of choice to prevent atherosclerotic cardiovascular disease (ASCVD) events following the release of the American College of Cardiology/American Heart Association (ACC/AHA)’s 2018 guidelines on the management of blood cholesterol [1]. This is because dyslipidemia is itself an independent risk factor for ASCVD [2].

Statin use for primary prevention among patients who do not show any signs of ASCVD is controversial [3–5]. A recent large-scale experimental study reported a 27% higher risk of new-onset diabetes following statin use [6], which has prompted the United States Food and Drug Administration to add a risk of new-onset diabetes to statin labels [7]. However, some studies support the hypothesis that the lipid-lowering effects of statins for secondary prevention among patients with ASCVD outweigh their adverse metabolic effects [8, 9]. Therefore, there is a need for further assessment of the risks of statin use in terms of new-onset diabetes, particularly considering the large number of patients in need of primary prevention of ASCVD and the widespread use of statins for that purpose [10].

The current study adds additional global epidemiological evidence of the effect of statin treatment on new-onset type 2 diabetes among individuals without cardiovascular disease using representative insurance claims data in South Korea. As epidemiological outcomes vary by demographic and socioeconomic circumstances, statins are not likely to pose the same degree of metabolic risk to all ethnicities. Indeed, the risk of new-onset diabetes associated with statin use is significantly higher in Asia [11, 12], where the prevalence of type 2 diabetes has increased twofold or more within the last decade [13]. Type 2 diabetes occurs even at a low BMI in Asia [14], leading to the development of a specialized approach for diabetes prevention and management becoming a top public health priority [14].

We also investigated the heterogeneity in the impact of statin use on new-onset type 2 diabetes by exposure period, intensities, and doses for all statins and by statin subtype. Despite its importance, there is no consensus regarding the intensity- and dose-dependent risk profile of statins with regard to new-onset diabetes. Given the potential increase in the use of intensive statin regimens based on the new ACC/AHA guidelines, it is important to conduct a detailed assessment of the association between statin use and new-onset type 2 diabetes.

## Methods

### Study design and population

We conducted a population-based retrospective cohort study using the National Health Insurance Service-National Sample Cohort. This dataset contains detailed data on the history of diagnoses and drug prescriptions as well as demographic and mortality-related information for 1,025,340 subjects from January 1, 2002 to December 31, 2013 [15]. The target subjects of this study were those aged 40 to 74 and newly diagnosed with dyslipidemia with or without statin prescriptions. We defined the index date as the date of the first statin prescription for statin users and the date of a hyperlipidemia diagnosis for non-users. To identify prior disease status, we set the first 3 years (2002–2004) as the pre-index period. The index period was defined as January 1, 2005 to December 31, 2005 to minimize immortal time bias.

For the final sample, we first retained the 389,880 subjects aged 40 to 74 in 2005 from among the 1,025,340 participants enrolled in 2002. We then retained 294,805 subjects after applying the following exclusion criteria based on assessments made before the index period, that is, during 2002–2004: (1) patients who had been diagnosed with any type of diabetes; (2) patients who were previously diagnosed with any major type of ASCVD, including coronary artery disease, cerebrovascular disease, and peripheral artery disease; (3) patients who were prescribed any statins; (4) patients who had been diagnosed with dyslipidemia; (5) patients with inpatient records with any type of cancer; and (6) patients who were diagnosed with liver cirrhosis, which can cause safety concerns that can discourage the use of statins (see Additional file 1: Table S1 for specific diagnosis codes). We further restricted our sample to those who were diagnosed with dyslipidemia in 2005, leaving only 17,074 subjects.

**Fig. 1:**
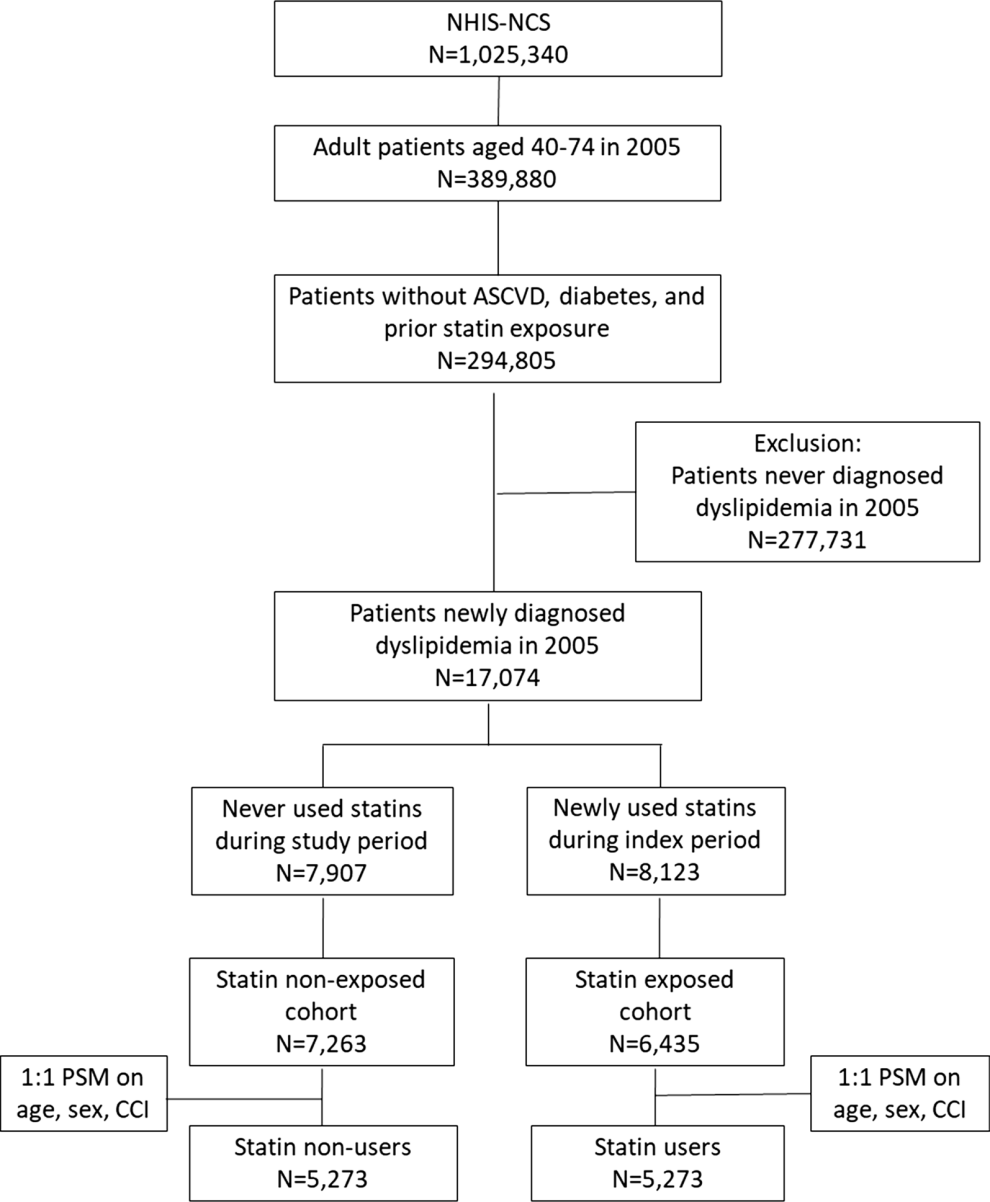
Selection and exclusion flowchart

We then dichotomized the sample into statin users (n = 8123) versus non-users (n = 7907). Statin users were further restricted to the 6435 subjects who received their first statin prescription during the index period, who had at least two statin prescriptions per 6 months during the study period [16], and who were diagnosed with diabetes after first statin use during the index year. Non-users were also further restricted to the 7263 patients who had at least one ambulatory care visit but had not received any statin prescriptions during the study period and who were not diagnosed with type 2 diabetes before their dyslipidemia diagnosis during the index period. We adopted the greedy nearest neighbor algorithm in propensity score matching [17] to match statin users with non-users in a 1:1 ratio, using age, sex, and the Charlson Comorbidity Index (CCI) score as matching variables. Propensity score matching is a well-established technique to address possible confounding by indication and to maintain a balance between exposure and non-exposure groups [17]. After propensity score matching, 5273 statin users and 5273 non-users were analyzed (Fig. 1).

### Assessment of exposure to statins

We identified atorvastatin, fluvastatin, lovastatin, rosuvastatin, simvastatin, pitavastatin, and pravastatin for analysis. Information on statin use was obtained from the dispensing data using the Anatomical Therapeutic Chemical classification (Additional file 1: Table S2). We categorized statin use into three levels according to duration: less than 2 years, 2 to 5 years, and more than 5 years. Statins were further classified according to their standardized lipid-lowering effect based on the defined daily dose (DDD), which is an assumed maintenance dose per day for a drug used for its main indication in adult patients as defined by the World Health Organization [18]. We also calculated cumulative DDD (cDDD) of statins per year by summing DDDs prescribed per year per individual, and further classified statin users by cDDD per year as follows: (1) less than 30 cDDD per year, that is, used less than two consecutive months per year considering that clinicians often prescribe less than 0.5 DDD of statins in South Korea; (2) 30–120 cDDD per year, that is, 2 to 8 months; (3) 120–180 cDDD per year, that is, 8 months to a year; and (4) over 180 cDDD per year, that is, a year of using over 1 DDD.

### Identification of new-onset type 2 diabetes

Follow-up for the target subjects ended when new-onset type 2 diabetes was initially identified or in the case of death. Otherwise, the endpoint of follow-up was set to December 31, 2013. The primary outcome of this study was incident cases of type 2 diabetes with International Classification of Disease, 10th revision diagnosis codes E11 and E14 (Additional file 1: Table S1). In a sensitivity analysis, patients who were diagnosed with new-onset type 2 diabetes in the first 1 month, 3 months, and 6 months after the index date were respectively removed from the sample to assess the possibility of surveillance bias. The incidence of type 2 diabetes was calculated as the number of patients with diabetes divided by the observed person-time.

### Covariate assessment

Covariates included demographic variables (age and sex) and socioeconomic status variables (insurance premium level and urbanization of residence), all of which were defined at the index date. Age was classified into seven categories: 40–44 (reference group), 45–49, 50–54, 55–59, 60–64, 65–69, and 70–74. The insurance premium level reported in deciles was determined by the household income and classified into six categories: 0–2 (reference group), 3–4, 5–6, 7–8, 9, and 10. The capital and six metropolitan cities were classified as urban and the rest as rural, with the latter as the reference. We also controlled for baseline comorbidities and co-medications identified from 2002 to 2004. Baseline comorbidities at the index date included hypertension, prediabetes, liver disease, chronic renal disease, family history of diabetes, morbid obesity, and other metabolic diseases (Additional file 1: Table S1). The number of outpatient and inpatient records from 2002 to 2004 were controlled for as indicators of prior clinical evaluation. We also included the CCI as a composite comorbidity score [19]. Concomitant medications considered to affect blood glucose level, including fibrates, niacin, ACE inhibitors, angiotensin II receptor blockers, calcium channel blockers, thiazide diuretics, and corticosteroids, were controlled for in all estimations [2–22] (Additional file 1: Table S2).

### Statistical analysis

We compared subjects’ baseline characteristics based on statin use (statin users versus non-users) using the *t* test for continuous variables and the Chi square test for categorical variables. We also assessed the appropriateness of matching using standardized difference statistics for matching (age, sex, and the CCI score) and other variables [23, 24]. Small (< 10%) absolute values of the standardized difference statistics support the assumption of balance between the treatment groups [25]. We calculated the overall incidence per 1000 person-years. We conducted time-to-event analyses using the Cox proportional hazards model to estimate the association between statin use and new-onset type 2 diabetes. The underlying time scale was days until type 2 diabetes onset from the index date. We tested the proportional hazards assumption with log–log survival curves and could not reject the proportionality in the risk over time. Therefore, we used extended Cox models instead of the basic Cox proportional hazards model to capture the changed hazard ratios (HRs) over the duration of statin use, which is time-varying based on the exposure duration in the model.

We estimated multivariate models adjusting for all of the aforementioned covariates as time-fixed factors (i.e., those measured at the index date). We generated adjusted Kaplan–Meier curves to compare the time to new-onset type 2 diabetes by statin use. We have also conducted an analysis of the diabetogenic risk of statins by statin subtype. Both unadjusted and adjusted HRs with 95% confidence intervals (CI) were calculated. All statistical analyses were performed with SAS version 9.4 (SAS Institute, Cary, NC).

## Results

### Characteristics of the study cohort

Table 1 shows the baseline characteristics of the final sample. The mean follow-up duration was 6.83 years among statin users and 7.37 years among non-users. The leading comorbidity among statin users was hypertension (60.14% of statin users; 40.85% of non-users) while among non-users it was liver disease (48.57% of statin users; 61.86% of non-users). The leading co-medication both in statin users and non-users was beta-blockers (24.73% of statin users; 16.73% of non-statin users). The majority of the sample had a CCI of 0 (72.65% of statin users; 71.91% of non-users). The mean cDDD of statins during the overall study period was 561.32 and the mean cDDD per year was 11.58. DDD and the cDDD per prescription by statin subtype based on the initial prescription are presented in Table 2.

**Table 1:** Baseline characteristics of the study population after propensity score matching

Variable	Patients, No (%)		p-value	The standardized difference statistics^a^
	Statin group (N = 5273)	Non-statin Group (N = 5273)		
Sex			< 0.0001	
Male	2288 (43.39)	2534 (48.06)		− 9.4
Female	2985 (56.61)	2739 (51.94)		9.4
Age at entry			0.2309	
40–44	544 (10.32)	551 (10.45)		− 0.4
45–49	905 (17.16)	920 (17.45)		− 0.8
50–54	1027 (19.48)	1006 (19.08)		1.0
55–59	1003 (19.02)	971 (18.41)		1.6
60–64	861 (16.33)	749 (14.20)		5.9
65–69	570 (10.81)	642 (12.18)		− 4.3
70–74	363 (6.88)	434 (8.23)		− 5.1
Insurance premium level			0.3051	
1	669 (12.69)	695 (13.18)		− 1.5
2	743 (14.09)	724 (13.73)		1.0
3	808 (15.32)	867 (16.44)		− 3.1
4	1179 (22.36)	1200 (22.76)		− 1.0
5	782 (14.83)	774 (14.68)		0.4
6	1092 (20.71)	1013 (19.21)		3.7
Urbanization of residence			0.0004	
Urban	2681 (50.84)	2499 (47.39)		6.9
Rural	2592 (49.16)	2774 (52.61)		− 6.9
Charlson comorbidity index score			0.1937	
0	3831 (72.65)	3792 (71.91)		1.7
1	545 (10.34)	588 (11.15)		− 2.6
2	762 (14.45)	732 (13.88)		1.6
≥ 3	135 (2.56)	161 (3.05)		− 3.0
Comorbidity				
Hypertension	3171 (60.14)	2154 (40.85)	< 0.0001	39.3
Prediabetes	217 (4.12)	248 (4.70)	0.1415	− 2.9
Liver disease	2561 (48.57)	3262 (61.86)	< 0.0001	− 27.0
Metabolic disorders	315 (5.97)	413 (7.83)	0.0002	− 7.3
Renal disease	648 (12.29)	782 (14.83)	0.0001	− 7.4
Hypothyroidism	261 (4.95)	361 (6.85)	< 0.0001	− 8.1
Family history of diabetes	10 (0.19)	9 (0.17)	0.8184	0.4
Morbid obesity	47 (0.89)	31 (0.59)	0.0690	3.5
Number of outpatient visits in the previous 3 years, mean (SD)	30.65 (21.92)	27.95 (20.33)	< 0.0001	7.1
Number of hospitalizations in the previous 3 years, mean (SD)	0.2712 (0.94)	0.3006 (0.91)	0.1038	− 2.4
Comedication				
Fibrates	208 (3.94)	117 (2.22)	< 0.0001	10
Niacin	26 (0.49)	28 (0.53)	0.7849	− 0.5
Corticosteroids	189 (3.58)	204 (3.87)	0.4406	− 1.5
Hypertension medications				
Beta-blocker	1304 (24.73)	882 (16.73)	< 0.0001	19.8
ACE inhibitor	533 (10.11)	295 (5.59)	< 0.0001	16.8
ARB	470 (8.91)	237 (4.49)	< 0.0001	17.7
CCB	1249 (23.69)	791 (15.00)	< 0.0001	22.1
Thiazide	1102 (20.90)	729 (13.83)	< 0.0001	18.8

*ARB* angiotensin receptor blocker, *CCB* calcium channel blocker

^a^Small (< 10%) absolute values of the standardized difference statistics support the assumption of balance between treatment groups

**Table 2:** Dose of statin by subtype based on the initial prescription

Statin subtype	Mean (SD)	
	DDD of statin per prescription	Cumulative DDD of statin per prescription
Total (5273)	0.667 (0.25)	11.58 (86.99)
Atorvastatin (1071)	0.61 (0.27)	13.09 (33.66)
Fluvastatin (59)	1.15 (0.31)	8.83 (12.79)
Lovastatin (771)	0.444 (0)	4.19 (5.71)
Pitavastatin (27)	1 (0)	13.41 (19.3)
Pravastatin (391)	0.61 (0.43)	6.32 (11.48)
Rosuvastatin (243)	1.03 (0.2)	10.84 (15.22)
Simvastatin (2711)	0.71 (0.19)	13.95 (119.13)

### Study outcomes

The cumulative incidence of type 2 diabetes is presented in Table 3. During follow-up, 3034 patients developed type 2 diabetes: 1871 statin users (61.67%) and 1163 non-users (38.33%). The overall incidence of type 2 diabetes was 40.52 per 1000 person-years (51.95 for statin users, 29.93 for non-users) (Table 3). When we defined diabetes more strictly as those prescribed anti-diabetic medication, the incidence of diabetes was reduced to 26.35 versus 10.09 per 1000 person‐years for the statin and non-statin groups, respectively (Additional file 1: Table S3).

**Table 3:** Cumulative incidence by statin use status

Variables	Statin group (N = 5273)	Non-statin group (N = 5273)
Follow-up period (years), mean (SD)	6.83 (2.82)	7.37 (2.51)
Incidence (persons)	1871	1163
Total person-years	36,015.19	38,856.24
Cumulative incidence rate (per 1 000 person-years), mean (± SD)	51.95 (36.77–88.49)	29.93 (22.32–45.38)

**Fig. 2:**
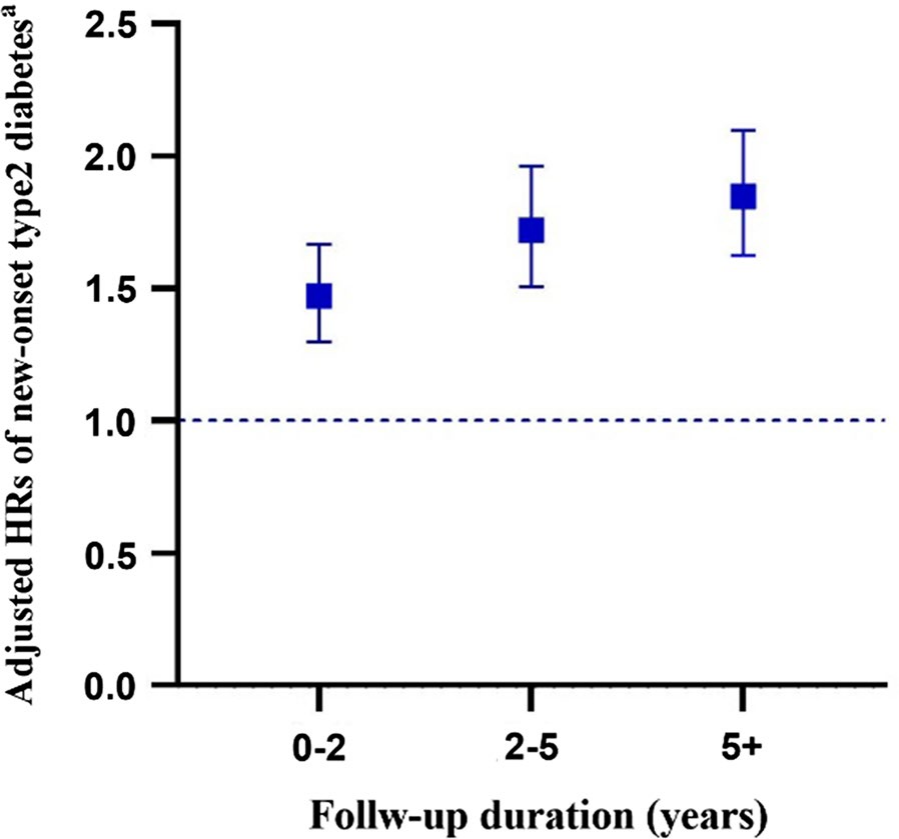
Time-varying hazard ratios (95% CI) of statin use compared to non-statin use. ^**a**^ Model was adjusted for age, sex, income level, urbanization of residence, Charlson comorbidity index, comorbidities (hypertension, prediabetes, liver disease, renal disease, metabolic disorder, family history of diabetes, obesity, hypothyroidism), number of outpatient visits in the previous 3 years, number of hospitalizations in the previous 3 years, comedications (fibrates, niacin, corticosteroids, hypertension medications). *p < 0.001. HR, hazard ratio; CI, confidence interval

The risk of type 2 diabetes increased both in the unadjusted and adjusted Cox regression models (adjusted HR = 1.47 [95% CI 1.3–1.67] for less than 2 years of exposure; 1.72 [1.51–1.96] for 2 to 5 years of exposure; and 1.85 [1.62–2.1] for over 5 years of exposure) (Additional file 1: Table S4; Fig. 2).

**Fig. 3:**
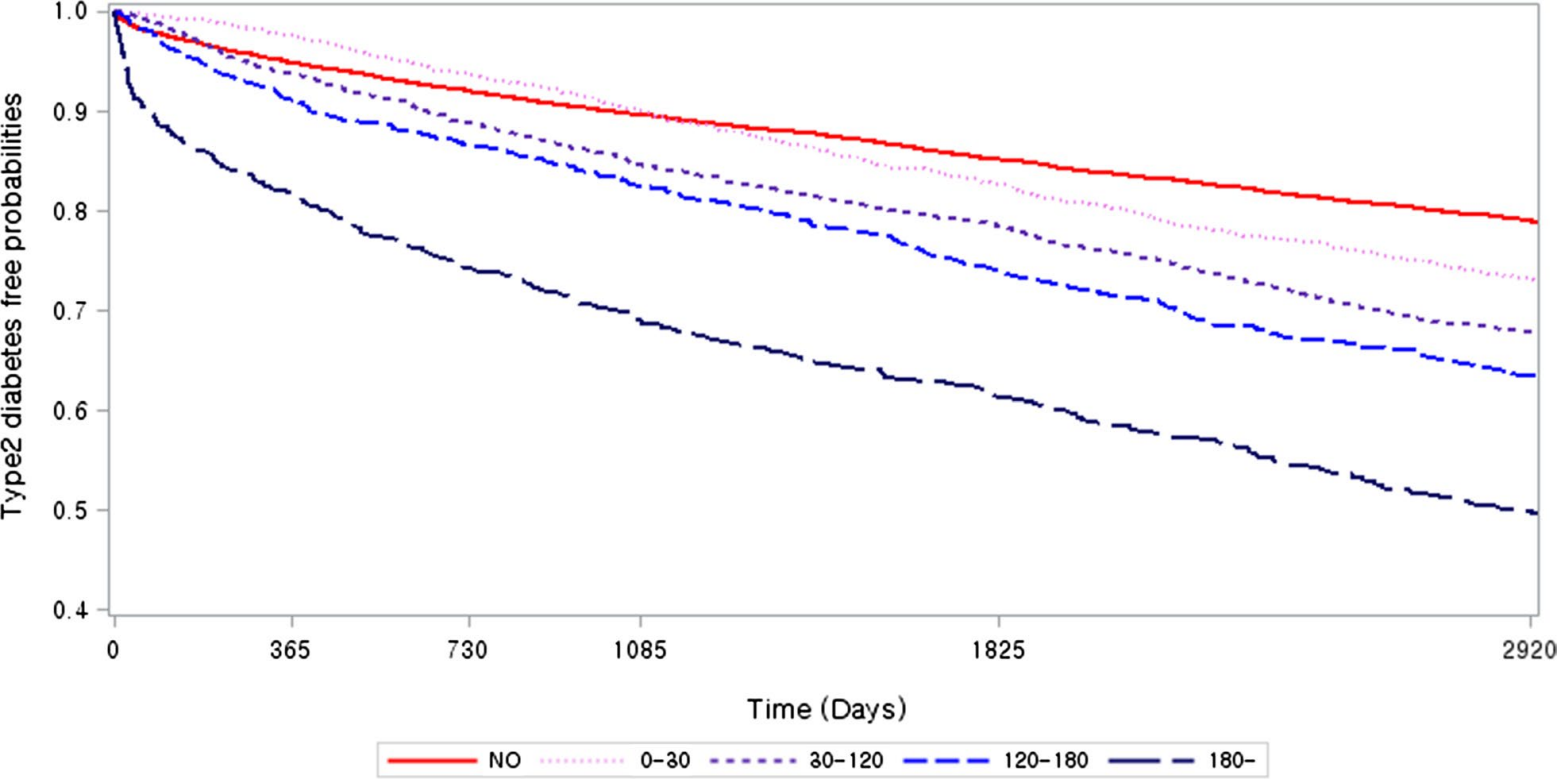
Kaplan-Meier curves according to cumulative daily defined dose (cDDD) per year

The next model assessed whether the mean cumulative statin dosage per year was associated with the risk of new-onset type 2 diabetes. Patients with higher statin maintenance doses had a higher risk of new-onset type 2 diabetes. The risk of new-onset type 2 diabetes differed among statin users according to cDDD per year (adjusted HR = 1.31 [95% CI 1.18–1.46] for less than 30 cDDD per year; 1.58 [1.43–1.75] for 30–120 cDDD per year; 1.83 [1.62–2.08] for 120–180 cDDD per year; and 2.83 [2.51–3.19] for more than 180 cDDD per year (Additional file 1: Table S5; Fig. 3).

The relationship did not substantially change and remained statistically significant regardless of whether we included subjects diagnosed with new-onset type 2 diabetes 1 month, 3 months, or 6 months from the index date. Taking the non-users as the reference, overall, the adjusted HRs of type 2 diabetes were higher in statin users, with upward patterns observed in groups with longer exposure periods. The results of the sensitivity analyses provided reassurance that our HRs were capturing a true association (Additional file 1: Table S6).

Further analyses by statin subtype indicate that the diabetogenic effect of pitavastatin was not statistically significant, but the risk was the largest for atorvastatin (HR = 1.942, 95% CI 1.661–2.269) followed by rosuvastatin (HR = 1.932, 95% CI 1.511–2.471). We have also added analyses of the impact of each statin subtype on T2D incidence by years of exposure. However, we noted that pitavastatin was prescribed for only 27 patients and the frequency for fluvastatin was only 59. Therefore, we have addressed the subgroup analyses by years of exposure except for pitavastatin and fluvastatin.

When the duration of exposure was grouped as three intervals (< 2 years, 2–5 years, and 5 years or longer), short-term statin exposure (< 2 years) was not associated with type 2 diabetes incidence in all statin subtypes explored. At the same time, long-term exposure (≥ 5 years) to statins was associated with a statistically significant increase in the risk of new onset type 2 diabetes in all statin subtypes explored, with the highest magnitude for simvastatin (HR = 1.916, 95% CI 1.647–2.228) followed by atorvastatin (HR = 1.830, 95% CI 1.487–2.252) (Table 4).

**Table 4:** Cox regression analysis on new-onset type2 DM according to years of exposure

(N)	Unadjusted Hazard Ratio^a^				Adjusted Hazard Ratio^a^			
	HR	95% CI		p-value	HR	95% CI		HR
Non-statin (5273)	1.000				1.000			
Atorvastatin (1071)	1.791	1.598	2.009	< 0.0001	1.942	1.661	2.269	1.942
< 2 years (137)	1.027	0.846	1.248	0.7853	0.989	0.8	1.222	0.989
2–5 years (127)	1.007	0.822	1.234	0.9448	1.005	0.791	1.277	1.005
≥ 5 years (807)	1.907	1.563	2.327	< 0.0001	1.83	1.487	2.252	1.83
Lovastatin (771)	1.847	1.624	2.101	< 0.0001	1.915	1.621	2.263	1.915
< 2 years (103)	1.07	0.862	1.328	0.5382	1.005	0.793	1.275	1.005
2–5 years (92)	1.113	0.885	1.401	0.3599	1.068	0.826	1.382	1.068
≥ 5 years (576)	1.942	1.551	2.431	< 0.0001	1.846	1.458	2.337	< 0.0.0001
Pravastatin (391)	1.662	1.39	1.986	< 0.0001	1.758	1.429	2.164	< 0.0001
< 2 years (42)	0.914	0.677	1.234	0.5582	0.981	0.706	1.363	0.9093
2–5 years (50)	1.256	0.946	1.666	0.1148	1.236	0.899	1.699	0.1911
≥ 5 years (299)	1.779	1.335	2.371	< 0.0001	1.633	1.211	2.203	0.0013
Rosuvastatin (243)	1.723	1.381	2.15	< 0.0001	1.932	1.511	2.471	< 0.0001
< 2 years (32)	0.931	0.653	1.328	0.6927	0.826	0.547	1.247	0.3635
2–5 years (30)	1.727	1.198	2.491	0.0034	1.712	1.133	2.586	0.0107
≥ 5 years (181)	1.578	1.053	2.365	0.0271	1.587	1.051	2.398	0.0282
Simvastatin (2711)	1.669	1.531	1.819	< 0.0001	1.794	1.567	2.055	< 0.0001
< 2 years (310)	0.93	0.802	1.078	0.3341	0.912	0.776	1.071	0.2617
2–5 years (290)	0.988	0.846	1.155	0.8804	0.979	0.824	1.163	0.8103
≥ 5 years (2111)	1.967	1.701	2.274	< 0.0001	1.916	1.647	2.228	< 0.0001

^a^Model was adjusted for age, sex, income level, urbanization of residence, Charlson comorbidity index, comorbidities (hypertension, prediabetes, liver disease, renal disease, metabolic disorder, family history of diabetes, obesity, hypothyroidism), number of outpatient visits in the previous 3 years, number of hospitalizations in the previous 3 years, comedications (fibrates, niacin, corticosteroids, hypertension medications). ^*^p < 0.001. HR, hazard ratio; CI, confidence interval

## Discussion

The present study showed that long-term statin use can induce new-onset type 2 diabetes in South Korean patients with dyslipidemia, using a nationally representative real-world dataset from insurance claims. Several large-scale experimental studies have reported an increased risk of new-onset diabetes in statin users, and a meta-analysis of randomized controlled trials (RCTs) confirmed this (9% increased risk of new-onset type 2 diabetes) [26–29]. However, the rigorous selection of subjects for RCTs may lead to the exclusion of individuals at higher risk of adverse events. In addition, while diabetes is a chronic condition that may not be easily identified, most existing RCTs concluded before many patients could be diagnosed. Also, these analyses based on RCTs were post hoc, and thus, application in the real-world setting is limited.

We showed that the estimated magnitude of the diabetogenic risk of statins is greater in South Koreans than in western populations. Although observational studies in Canada, Italy, Ireland, and New Zealand have confirmed a significant increase in statin-induced diabetes [30–33], it is difficult to apply these results to Asia. Considering the different pharmaco-epidemic responses and characteristics of diabetes in Asians, the risk of new-onset diabetes following long-term statin use is likely to be heterogeneous among Asians. Furthermore, most previous studies on Asians have only used a single hospital center or health screening data that lacked external validity [11, 12, 34–36].

A recent study based on the national insurance claims data in South Korea showed that adults without diabetes at increased risk of coronary artery diseases had a modestly higher risk of incident diabetes after the initiation of statin therapy [37]. Similar to the Go (2020)’ study [37], we estimated the risk of new-onset type 2 diabetes based on the database of national insurance claims, where all South Koreans are compulsory beneficiaries and all medical providers are compulsory providers [15]. Therefore, our data reflect a representative sample of the national population and our results possess strong external validity.

Statins have demonstrated a heterogeneous potential to increase the incidence of diabetes [38, 39]. A previous meta-analysis of RCTs regarding the primary prevention of ASCVD demonstrated a relatively low risk of new-onset type 2 diabetes [40]. However, the risk of type 2 diabetes was statistically significant even after a relatively short period of statin exposure of a mean 254 days and median 1.9 years [26, 41]. The small absolute risk of diabetes is outweighed by cardiovascular benefits in the short and medium term in individuals for whom statin therapy is recommended; long-term risk, however, has not fully been assessed in the previous literature [40]. Our study expands the literature by estimating the time-dependent hazard levels to address the time-exposure interaction in the effect of statin use on new-onset type 2 diabetes. Our study showed that the inductive effect of new-onset type 2 diabetes is enhanced by the cumulative dose of statins per year. Short-term statin exposure (< 2 years) was not associated with new onset type 2 diabetes in all statin subtypes, whereas long-term exposure (≥ 5 years) to statins was associated with a statistically significant increase in the risk of new onset type 2 diabetes in all statin subtypes.

Previous studies also have reported heterogeneous diabetogenic effects of statins by their subtypes [42]. For example, atorvastatin has been mostly reported to be more diabetogenic, whereas pitavastin is less so [43–45] or not diabetogenic [46]. Another study showed that even high-dose pitavastatin did not increase the risk of new onset of diabetes in patients at a high risk of developing diabetes during a 3-year follow-up [47]. We have addressed such variations not only for the overall use by statin subtypes but also by cumulative dosages per year and years of exposure for each subtype. Our findings are consistent with the previous studies in that pitavastatin did not show a statistically significant diabetogenic effect, whereas such an effect was the largest for atorvastatin followed by rosuvastatin.

Our study focused specifically on type 2 diabetes because that is the form clearly attributable to statin use. We also note that our identification of type 2 diabetes applies to a broad spectrum of severity, including mild diabetes without anti-diabetic medications. Most cases of incident diabetes in older patients are likely to be type 2, and thus, we excluded patients aged under 40. Our restrictive design, part of a conservative approach, is likely to yield more robust outcomes than other approaches. Patients who are prescribed statins are more likely to be clinically examined or treated before the onset of type 2 diabetes, and thus have a higher chance of being diagnosed with type 2 diabetes than dyslipidemia patients without statin prescriptions. However, we adjusted for the frequency of outpatient visits to control for such detection bias.

The data used for the present study, which were extracted from insurance claims, have caveats. Our data did not include blood lipid profiles, fasting glucose levels, or hemoglobin A1c levels. Previous studies have suggested that low LDL cholesterol is associated with the onset of type 2 diabetes independent of statin use [48]. Some researchers have claimed that epicardial adipose tissue thickness at systole is a consistent independent predictor of new-onset diabetes mellitus in patients with coronary artery disease treated with high-intensity statins [49]. On the contrary, another study reported that even though statin therapy improved the lipid profile it had no effect on epicardial adipose tissue [50].

We also could not control for potential confounding factors such as BMI, health behaviors (e.g., smoking status, alcohol consumption, physical activity, or dietary intake), and genetic factors. A meta-analysis reported an increased metabolic risk in cases of LDL-C-lowering genetic variants [51]. However, we conducted propensity score matching to minimize selection bias. Considering the complexity of the relationships of metabolic diseases such as dyslipidemia, hypertension, and type 2 diabetes [52], we evaluated the effect of statin use on new-onset type 2 diabetes, controlling for dyslipidemia status and adjusting for hypertension. We also adjusted for a wide range of covariates including socioeconomic, demographic, and clinical factors, which have been reported to moderate the relationship between statin use and new-onset type 2 diabetes [53, 54].

Caution should be used when applying our results to patients with different ASCVD risk profiles or histories because we measured risk of new-onset type 2 diabetes only for patients taking statins for primary prevention of ASCVD. Statin use for primary prevention of ASCVD is recommended to be based on individual decisions [1]. It has also been suggested that statin-dependent type 2 diabetes might be prognostically less adverse than diabetes unlikely to be induced by statins [30]. Future studies with longer follow-ups based on a prospective cohort study would further advance our understanding of the potential risks and benefits of stains for primary prevention of ASCVD.

## Conclusions

The current study confirms the need for rigorous monitoring of patients taking statins, particularly those with established risk factors for diabetes onset. Our findings also suggest the necessity of taking the diabetogenicity of statins into consideration in clinical practice, emphasizing the concomitant need for dietary control and exercise.

## Supplementary information

**Additional file 1.** Additional tables.

## Data Availability

The datasets used and/or analyzed during the current study are available from the corresponding author on reasonable request.

## Abbreviations

ASCVD: Atherosclerotic cardiovascular disease
ACC/AHA: American College of Cardiology/American Heart Association
CCI: Charlson comorbidity index
DDD: Defined daily dose
cDDD: Cumulative defined daily dose
HR: Hazard ratio
RCTs: Randomized controlled trials
