# Supplementary material for: Time-varying and dose-dependent effect of long-term statin use on risk of type 2 diabetes: a retrospective cohort study
Source: Cardiovasc Diabetol. 2020 May 16;19:67. doi: 10.1186/s12933-020-01037-0 (PMC7231413; doi:10.1186/s12933-020-01037-0)
Supplement: Supplementary file 1 — Additional file 1. Additional tables. [file 12933_2020_1037_MOESM1_ESM.docx]

Table S1. ICD-10 codes of diagnoses used for defining the study population, comorbidities, and outcomes

| Diagnosis | ICD-10 codes^¶^ |
| --- | --- |
| Study population selection |  |
| Any type of diabetes | E10, E11, E12, E13, E14 |
| Atherosclerotic cardiovascular disease (ASCVD) |  |
| Cerebrovascular disease | I60, I61, I62, I63, I65, I66, I67, I68, I69, G45, G46 |
| Peripheral vascular disease | I70 |
| Acute myocardial infarction | I20, I21, I22, I23, I24, I25 |
| Stroke | I64 |
| Congestive heart failure | I50 |
| Dyslipidemia | E78 |
| Any type of cancer | C00-C97 |
| Liver cirrhosis | K74 |
| Comorbidities |  |
| Hypertension | I10-I15 |
| Prediabetes | R73, R81, Z131 |
| Liver disease | K70-K77 / B15-B19 |
| Family history of DM | Z833 |
| Metabolic disorders except for dyslipidemia | E70-E90 (except for E78) |
| Morbid obesity | E66 |
| Chronic renal disease | N00, N01, N02, N03, N04, N05, N06, N07, N08, N10, N11, N12, N13, N14, N15, N16, N17, N18, N19, N25, N26, Z940, Z992 |
| Hypothyroidism | E02, E03 |
| Outcome |  |
| Type 2 diabetes | E11, E14 |

^¶^International Classification of Disease, 10th revision by the World Health Organization

Table S2. Anatomical Therapeutic Chemical (ATC) codes of medications used in the study

| International Nonproprietary Name (INN) | ATC codes^§^ |
| --- | --- |
| Study medications |  |
| Atorvastatin | C10AA05, C10BX08, C10BX03, C10BA05, C10BX15, C10BX12, C10BX06, C10BX11 |
| Fluvastatin | C10AA04 |
| Lovastatin | C10AA02, C10BA01 |
| Rosuvastatin | C10AA07, C10BX05, C10BX09, C10BA06, C10BX10, C10BX07, C10BX14, C10BX13 |
| Simvastatin | C10AA01, C10BX01, C10BA02, C10BA04, C10BX04 |
| Pitavastatin | C10AA08 |
| Pravastatin | C10AA03, C10BX02, C10BA03 |
| Comedications |  |
| Fibrates | C10AB01, C10AB02, C10AB03, C10AB04, C10AB05, C10AB06, C10AB07, C10AB08, C10AB09, C10AB10, C10AB11 |
| Corticosteroids | D07AA01, D07AA02, D07AA03, D07AB01, D07AB02, D07AB03, D07AB04, D07AB05, D07AB06, D07AB07, D07AB08, D07AB09, D07AB10, D07AB11, D07AB19, D07AB21, D07AB30, D07AC**, D07AD** |
| Niacin | C10AD01, C10AD02, C10AD03, C10AD04, C10AD05, C10AD06, C10AD52 |
| Beta-blocker | C07AA01, C07AA02, C07AA03, C07AA05, C07AA06, C07AA07, C07AA12, C07AA15, C07AA19, C07AB02, C07AB03, C07AB04, C07AB05, C07AB07, C07AB09,  C07AB12, C07AG01, C07AG02, C07BA68, C07BB02, C07BB03, C07CA03, C07DA06 |
| ACE inhibitor | C09AA01, C09AA03, C09AA02, C09AA09, C09AA05, C09AA04, C09BA04, C09AA06, C09AA08, C09AA07, C09BB05, C09BA01, C09AA16, C09BB, C09BA02 |
| ARB | C09DA07, C09CA06, C09CA01, C09DA08, C09CA03, C09DA01, C09CA04, C09DA03, C09DB01, C09CA07, C09CA08, C09DA04, C09CA02, C09DA06, C09DX01,  C09DX03, C09DB04, C09DB02, C09CA09 |
| CCB | C08CA01, C08CA02, C08CA03, C08CA04, C08CA05, C08CA06, C08CA07, C08CA08, C08CA09, C08CA12, C08CA13, C08CA15 |
| Thiazide | C03AA03, C03AA06, C03AA91, C03AA07 |

^§^Anatomical Therapeutic Chemical codes by the World Health Organization. ARB, angiotensin receptor blocker; CCB, calcium channel blocker.

Table S3. Cumulative incidence by statin use considering additional diagnostic criteria

| Variables | Statin group  (N = 5,273) | Non-statin Group  (N = 5,273) |
| --- | --- | --- |
| Follow-up period (years), mean (SD) | 6.83 (2.82) | 7.37 (2.51) |
| Incidence (persons) | 909 | 364 |
| Total person-years | 36015.19 | 38856.24 |
| Cumulative incidence (per 1000 person-years) | 25.24  (17.86–42.99) | 9.37  (6.99–14.2) |

Table S4. Cox regression analysis of new-onset type2 diabetes by duration of statin use

| Variable  (N) | Unadjusted HR | | Adjusted HR^a^ | |
| --- | --- | --- | --- | --- |
|  | HR | 95% CI | HR | 95% CI |
| Non-statin (5,273)  Statin (5,273) | 1.00 (Reference) | | 1.00 (Reference) | |
| ≤ 2 years | 1.52^*^ | 1.34–1.71 | 1.47^*^ | 1.3–1.67 |
| 2–5 years | 1.77^*^ | 1.56–2.02 | 1.72^*^ | 1.51–1.96 |
| ≥ 5 years | 1.91^*^ | 1.68–2.16 | 1.85^*^ | 1.62–2.1 |

**^a^** Model was adjusted for age, sex, income level, urbanization of residence, Charlson comorbidity index, comorbidities (hypertension, prediabetes, liver disease, renal disease, metabolic disorder, family history of diabetes, obesity, hypothyroidism), number of outpatient visits in the previous 3 years, number of hospitalizations in the previous 3 years, comedications (fibrates, niacin, corticosteroids, hypertension medications). ^*^p < 0.001. HR, hazard ratio; CI, confidence interval.

Table S5. Cox regression analysis of new-onset type2 diabetes by cDDD per year of statin use

| Variable  (N) | Cumulative incidence rate  (per 1000 person-years) | | Unadjusted HR | | Adjusted HR^a^ | | |  |
| --- | --- | --- | --- | --- | --- | --- | --- | --- |
|  | Mean (±SD) | | HR | 95% CI | | HR | 95% CI | |
| Non-statin (5,273) | 29.93 (22.32-45.38) | 1.00 (Reference) | | | 1.00 (Reference) | | |  |
| Statin (5,273)  cDDD/year |  |  | |  | |  |  | |
| ≤ 30 | 38.97 (29.53–57.22) | 1.3^*^ | | 1.17–1.44 | | 1.32^*^ | 1.19–1.47 | |
| 30 – 120 | 48.96 (35.03–81.39) | 1.62^*^ | | 1.47–1.79 | | 1.58^*^ | 1.43–1.75 | |
| 120 – 180 | 58.71 (40.7–105.47) | 1.94^*^ | | 1.72–2.19 | | 1.84^*^ | 1.62–2.09 | |
| > 180 | 91.78 (56.84–237.27) | 3.01^*^ | | 2.68–3.37 | | 2.85^*^ | 2.53–3.22 | |

**^a^** Model was adjusted for age, sex, income level, urbanization of residence, Charlson comorbidity index, comorbidities (hypertension, prediabetes, liver disease, renal disease, metabolic disorder, family history of diabetes, obesity, hypothyroidism), number of outpatient visits in the previous 3 years, number of hospitalizations in the previous 3 years, comedications (fibrates, niacin, corticosteroids, hypertension medications). ^*^p < 0.001. cDDD, cumulative defined daily dose; HR, hazard ratio; CI, confidence interval.

Table S6. Sensitivity analysis: Cox regression analysis on new-onset type 2 DM by statin use considering protopathic bias

| Variable (N) | Adjusted HR^a^ | |
| --- | --- | --- |
|  | HR | 95% CI |
| Patients with incident case within 1 month excluded (10,420) |  |  |
| Non-statin | 1.00 (Reference) | |
| Statin |  |  |
| ≤ 2 years | 1.54^*^ | 1.35–1.76 |
| 2–5 years | 1.72^*^ | 1.51–1.97 |
| ≥ 5 years | 1.85^*^ | 1.63–2.1 |
| Patients with incident case within 3 months excluded (10,291) |  |  |
| Non-statin | 1.00 (Reference) | |
| Statin |  |  |
| ≤ 2 years | 1.6^*^ | 1.39–1.86 |
| 2–5 years | 1.72^*^ | 1.5–1.96 |
| ≥ 5 years | 1.85^*^ | 1.62–2.1 |
| Patients with incident case within 6 months excluded (10,155) |  |  |
| Non-statin | 1.00 (Reference) | |
| Statin |  |  |
| ≤ 2 years | 1.59^*^ | 1.36–1.87 |
| 2–5 years | 1.72^*^ | 1.51–1.96 |
| ≥ 5 years | 1.85^*^ | 1.63–2.1 |

**^a^** Model was adjusted for age, sex, income level, urbanization of residence, Charlson comorbidity index, comorbidities (hypertension, prediabetes, liver disease, renal disease, metabolic disorder, family history of diabetes, obesity, hypothyroidism), number of outpatient visits in the previous 3 years, number of hospitalizations in the previous 3 years, comedications (fibrates, niacin, corticosteroids, hypertension medications). ^*^p < 0.001. HR, hazard ratio; CI, confidence interval.
